# Obesity, Inflammation, and Exercise Training: Relative Contribution of iNOS and eNOS in the Modulation of Vascular Function in the Mouse Aorta

**DOI:** 10.3389/fphys.2016.00386

**Published:** 2016-09-07

**Authors:** Josiane F. Silva, Izabella C. Correa, Thiago F. Diniz, Paulo M. Lima, Roger L. Santos, Steyner F. Cortes, Cândido C. Coimbra, Virginia S. Lemos

**Affiliations:** ^1^Department of Physiology and Biophysics, Institute of Biological Sciences, Universidade Federal de Minas GeraisBelo Horizonte, Brazil; ^2^Department of Physiological Science, Universidade Federal do Espírito SantoEspírito Santo, Brazil; ^3^Department of Pharmacology, Institute of Biological Sciences, Universidade Federal de Minas GeraisBelo Horizonte, Brazil

**Keywords:** obesity, vascular dysfunction, exercise training, iNOS, eNOS, TNFα

## Abstract

**Background:** The understanding of obsesity-related vascular dysfunction remains controversial mainly because of the diseases associated with vascular injury. Exercise training is known to prevent vascular dysfunction. Using an obesity model without comorbidities, we aimed at investigating the underlying mechanism of vascular dysfunction and how exercise interferes with this process.

**Methods:** High-sugar diet was used to induce obesity in mice. Exercise training was performed 5 days/week. Body weight, energy intake, and adipose tissues were assessed; blood metabolic and hormonal parameters were determined; and serum TNFα was measured. Blood pressure and heart rate were assessed by plethysmography. Changes in aortic isometric tension were recorded on myograph. Western blot was used to analyze protein expression. Nitric oxide (NO) was evaluated using fluorescence microscopy. Antisense oligodeoxynucleotides were used for inducible nitric oxide synthase isoform (iNOS) knockdown.

**Results:** Body weight, fat mass, total cholesterol, low-density lipoprotein cholesterol fraction, insulin, and leptin were higher in the sedentary obese group (SD) than in the sedentary control animals (SS). Exercise training prevented these changes. No difference in glucose tolerance, insulin sensitivity, blood pressure, and heart rate was found. Decreased vascular relaxation and reduced endothelial nitric oxide synthase (eNOS) functioning in the SD group were prevented by exercise. Contractile response to phenylephrine was decreased in the aortas of the wild SD mice, compared with that of the SS group; however, no alteration was noted in the SD iNOS^−/−^ animals. The decreased contractility was endothelium-dependent, and was reverted by iNOS inhibition or iNOS silencing. The aortas from the SD group showed increased basal NO production, serum TNFα, TNF receptor-1, and phospho-IκB. Exercise training attenuated iNOS-dependent reduction in contractile response in high-sugar diet–fed animals, decreased iNOS expression, and increased eNOS expression.

**Conclusion:** Obesity caused endothelium dysfunction, TNFα, and iNOS pathway up-regulation, decreasing vascular contractility in the obese animals. Exercise training was an effective therapy to control iNOS-dependent NO production and to preserve endothelial function in obese individuals.

## Introduction

Obesity is an important health problem and is closely associated with the onset and development of cardiovascular and metabolic diseases (Ponce-Garcia et al., 2015). Several studies have shown that the probability of cardiovascular disease occurrence is four times lower in a normal-weight individual (body mass index ≤ 25 kg.m^−2^) than in an obese person (body mass index ≥ 30 kg.m^−2^; Malik et al., 2004; Gil-Ortega et al., 2010; Wang et al., 2010). A chronic low-grade inflammation process in both the circulation and adipose tissue is a feature strictly associated with obesity (Hajer et al., 2008; Stapleton et al., 2008).

In obese subjects, vascular injury usually happens (Hajer et al., 2008; Stapleton et al., 2008). The on set and progress of vascular dysfunction are complex and could be related to several alterations, such as insulin resistance (Zecchin et al., 2007), hypertension (Li et al., 2007; Tang and Vanhoutte, 2008) and oxidative stress (Gil-Ortega et al., 2014). Together, these disorders can contribute to a reduction in the production and/or bioavailability of nitric oxide (NO), an important endothelium-dependent relaxant factor, leading to vascular dysfunction (Marchesi et al., 2009; Tziomalos et al., 2010; Davel et al., 2011; Fernández-Alfonso et al., 2013). NO reduction is commonly associated with a decrease in the expression and/or activity of endothelial nitric oxide synthase (eNOS; Stapleton et al., 2008; Marchesi et al., 2009). Although some studies have shown that up-regulation of the inducible nitric oxide synthase isoform (iNOS) expression is associated with obesity (Yang and Chen, 2003; Noronha et al., 2005), little data addressing the role of iNOS/NO pathway in the modulation of vascular contractile response in obese subjects exist.

The existence of comorbidities, such as hypertension and diabetes, has brought more complexity to the understanding of obesity-related vascular dysfunction (Hajer et al., 2008; Stapleton et al., 2008; Molica et al., 2014). Thus, the precise role played by obesity *per se* and that played by diseases associated with vascular injury in obese subjects remain unclear.

Regular physical activity has been considered a non-pharmacological therapy to treat obesity, reducing weight and improving vascular function (Kojda and Hambrecht, 2005). Vascular function improvement is typically related to a decrease in pro-inflammatory cytokines (Baynard et al., 2012; Linden et al., 2014) and reactive oxygen species (ROS) formation (Braga et al., 2015). Additionally, exercise training increases eNOS-derived NO production (Boa et al., 2014). Nonetheless, the effect of exercise training on iNOS pathway modulation in the blood vessels is unclear.

In this study, we used a mouse model of high-sugar diet-induced obesity without insulin resistance, diabetes, or hypertension. Here we present new insights on the signaling pathway involved in obesity-related vascular dysfunction and how exercise training prevent this alteration. This study provides evidence that (i) obesity causes TNFα-dependent iNOS up-regulation, which increases basal NO production and contributes to impaired aortic function, and (ii) exercise training reduces TNFα/TNF-receptor/NF-κB signaling activation, which differently modulates the imbalance between eNOS and iNOS expression in the aorta under normal and high caloric intake.

## Materials and methods

### Animals and diet

Experimental protocols were performed in accordance with the guidelines for the use and care of laboratory animals and were approved by the Ethics Committee for Animal Use (CEUA) of the Federal University of Minas Gerais (protocol #157/11). Twelve-week-old male C57BL/6J mice, obtained from CEBIO/ICB (UFMG, Brazil), were housed in a temperature-controlled room (24.0 ± 2.0°C) and maintained on a 12–12-h light/dark cycle with free access to water and food. The mice were randomly distributed into the following groups: sedentary mice fed a standard chow diet (SS), sedentary mice fed a high-sugar diet (SD), trained mice fed a standard chow diet (TS), and trained mice fed a high-sugar diet (TD). In some experiments we used age-matched iNOS^−/−^ mice (originally from Jackson Laboratories and bred at the Gnotobiology facility/ICB), as follows: iNOS^−/−^ mice fed a standard chow diet (iNOS^−/−^ standard chow) and iNOS^−/−^ mice fed a high-sugar diet (iNOS^−/−^ high-sugar diet).

The high-sugar diet consisted of 33% standard chow (Nuvilab®CR1; Nuvital, Brazil), 33% condensed milk and 7% sucrose by weight (the remaining is water; de Queiroz et al., 2012). The energy density was 12.26 kJ/g for the standard chow diet and 13.35 kJ/g for the high-sugar diet. The animals were fed with the diets for 8 weeks.

### Graded treadmill exercise test

Exercise capacity, estimated by running workload, was evaluated with a fatiguing exercise protocol (Rolim et al., 2007). After being adapted to a motor treadmill (Gaustec, Contagem, Brazil) over 1 week (10 min of exercise session), the mice were subjected to a workload running test; the speed being increased 3 m/min every 3 min from an initial velocity of 6 m/min with 5 grade of inclination until fatigue. This test determined the pre-exercise training maximal performance and the speed of the training protocol. Fatigue was defined as the point at which the animals were no longer able to maintain pace with the treadmill for at least 10 s. The test was repeated at the end of the fourth and 8th week of training to adjust the running speed and assess improvement in running performance. Workload (W) was calculated as W = [body weight (kg)] × [time to fatigue] × [treadmill speed (m/min)] × [sin θ (treadmill inclination)] (Soares et al., 2003).

### Exercise training protocol

Exercise training was performed over 8 weeks, simultaneous with the diet, 5 days/week (Rolim et al., 2007). The running speed and exercise duration were increased progressively to elicit 60% of maximal speed, achieved during a graded treadmill exercise test, for 60 min at the 4th week. This intensity was adjusted after the second graded treadmill exercise test and maintained during the rest of the training period. The untrained mice were exposed to treadmill exercise (5 min) twice a week to reduce the effects of treadmill exercise and handling stress.

### Body weight and metabolic parameters

Body weight gain and energy intake were evaluated during the 8 weeks of diet and exercise training. The energy intake was measured as follows: amount of food intake × energy density of the diets (de Queiroz et al., 2012). The animals were euthanized under fed conditions. The periaortic, epididymal and retroperitoneal adipose tissues were dissected, weighed, and stored at −80°C. The fat deposit weight was calculated as follows: adipose tissue weight/animal body weight × 1000. Serum concentration of glucose, triglycerides, total cholesterol, high-density lipoprotein cholesterol fraction (cHDL), and free fatty acids were measured using specific enzymatic kits (DOLES®, Brazil). The low-density lipoprotein cholesterol fraction (cLDL) concentrations were estimated by Friedewald equation (Friedewald et al., 1972). Serum leptin and insulin were measured by radioimmunoassay (Millipore®, St. Charles, MO, USA). The enzyme-linked immunosorbent assay (ELISA) kits (R&D System®, Minneapolis, USA) were used to measure MCP-1 and TNFα in serum and adipose tissues.

### Glucose tolerance and insulin sensitivity tests

Glucose oral tolerance test was performed in overnight-fasted mice at the end of the 8 weeks of treatment (Santos et al., 2013). Blood samples were obtained at 0, 15, 30, 45, and 60 min from the tail, after glucose (2 mg/g body weight) administration by gavage. For the insulin sensitivity test, an intraperitoneal administration of insulin (0.75 units/kg body weight; Sigma-Aldrich®, St. Louis, USA) was performed in overnight-fed mice. Tail blood samples were taken at 0, 15, 30, 60, 90, and 120 min after the injection for blood glucose level measurement (Santos et al., 2013). Glucose was measured using an Accu-Check glucometer (Roche Diagnostics®, Indianapolis, USA).

### Systolic blood pressure and heart rate assessments

Systolic blood pressure and heart rate were assessed by tail plethysmography (Silva et al., 2013). Before the protocol onset, the animals passed through a period of adaptation to the retainer. Data were registered by a plethysmograph (RTBP 2000, Kent Scientific®, USA) connected to a computer with WinDaq Data Acquisition software (Dataq Instruments, USA).

### Vascular function studies

Rings from the thoracic aorta (3.0 mm length) were mounted in an organ bath system containing *Krebs-Henseileit* solution (in mM): 118 NaCl, 4.7 KCl, 25 NaHCO_3_, 11 glucose, 1.2 KH_2_PO_4_, 1.2 MgSO_4_, 2.5 CaCl_2_, and pH 7.4 (Capettini et al., 2010). Isometric tension was recorded by transducers (World Precision Instruments, Inc., USA) connected to an amplifier-recorder (TBM-4 model; World Precision Instruments, Inc., USA) and a computer with an analog-to-digital converter board (AD16JR; World Precision Instruments, Inc.USA). WinDaq Data Acquisition software was used for data acquisition and analysis. Cumulative concentration-response curves to acetylcholine, phenylephrine or KCl were constructed in the vessels from the different groups in the presence or absence of different drugs. To evaluate the participation of nitric oxide synthase (NOS) in vascular function, we used L-NAME (300 μM), a non-selective NOS inhibitor; L-NNA (1 μM) at the concentration used, a selective inhibitor of eNOS; and L-NIL (10 μM), a selective inhibitor of iNOS. Ibuprofen (10 μM), a non-selective inhibitor of cyclooxygenase, was used to evaluate the participation of prostaglandins.

### Antisense oligodeoxynucleotides

We used the antisense oligodeoxynucleotide (AS-ODN) technique to silence iNOS as previously described (Capettini et al., 2008) with adaptations. The 16-base phosphorothioated AS-ODN were designed based on the mouse iNOS sequence. The sequence used was 5′-CACCTCCAACACAAGA-3′ (Genbank NM-010927.3). A phosphorothioated mismatch ODN (MM-ODN) sequence with the base composition 5′-GTCTTGAACTTCCCGATCT-3′ was used as control. Both sequences were purchased from Eurogentec North America Inc (San Diego, CA, USA). Animals received 2 nmol AS-ODN or MM-ODN, 12 h before the sacrifice. The efficiency of the AS-ODN to block iNOS expression was evaluated by Western blot (Supplementary Figure 1).

### Western blot

Western blot was performed as previously described (Capettini et al., 2008) with modifications. Briefly, 50 μg of total protein was separated in denaturing SDS/7.5% polyacrylamide gel. Proteins were electrotransferred onto a polyvinylidene fluoride membrane (Immobilon-P; Millipore, MA, USA). Membranes were incubated overnight with a specific primary antibody: mouse monoclonal anti-iNOS (1:1000), mouse monoclonal anti-eNOS (1:1000), goat polyclonal anti-phospho-Ser1177-eNOS (1:500), mouse monoclonal anti-TNF-R1 (1:1000), mouse monoclonal anti-p65 subunit NF-κB (1:1 000), rabbit polyclonal anti-IκB-α (1:1000), mouse monoclonal anti-phospho-Ser32-IκB-α (1:500), or mouse monoclonal anti-β-actin (1:3000). All antibodies were from Santa Cruz Biotechnology, Inc. (Santa Cruz, CA, USA). Subsequently, the membranes were incubated with a specific HRP-conjugated secondary antibody. Immunocomplexes were detected by chemiluminescence reaction (ECL® kit; Amersham, Les Ulis, France), and densitometric analysis was performed with ImageJ 1.46 software.

### Nitric oxide and superoxide detection

NO and superoxide production in the aorta was measured using fluorescent dyes: 4-amino-5-methylamino-2′, 7′-difluorescein diacetate (DAF-FM; 8 μM; Invitrogen, USA) and dihydroethidium (DHE; 5 μM; Invitrogen), respectively (Silva et al., 2016). Aortic rings were embedded in a freezing medium to obtain transverse sections (10 μm). After 10 min of incubation in a phosphate buffer (0.1 mM, pH = 7.4) containing CaCl_2_ (0.45 mM) at 37°C, the slices were incubated in a light-protected chamber with the respective dyes. Some aortic slices were incubated with Tiron (100 μM), a superoxide anion scavenger. The slices were mounted with DAPI/UltraCruz® Mounting Medium (Santa Cruz Biotechnology, Inc., CA, USA) and digital images were obtained using a fluorescence microscope (Eclipse Ti, Tokyo, Nikon, Japan) using a standard fluorescein filter with a 60 × objective. The images were analyzed using ImageJ 1.46 software by measuring the mean optical fluorescence density.

### Statistical analysis

Data are expressed as mean ± SEM. Concentration-response curves were compared using the area under the curve and the maximal reponse (E_max_) values in the different experimental conditions. The delta of the area under the curve (ΔAUC) was calculated as the difference between the concentration-response curves in the presence and the absence of different drugs. Two-way ANOVA with Tukey's multiple comparisons test was used to compare AUC, ΔAUC, and E_max_-values. One-way ANOVA followed by Tukey *post-hoc* test was used in the other experiments. Statistical analyses were performed using the GraphPad Prism software 7.00, and *p* < 0.05 was considered significant.

## Results

### General parameters

After 8 weeks of feeding with high-sugar diet, The SD group had ~50% greater calorie intake values and greater weight gain and epidydimal and periaortic fat mass than the SS group (Table 1). Exercise training attenuated the increase in epididymal fat and prevented weight gain and increase in periaortic fat mass. Furthermore, the diet induced a rise in serum cLDL levels, total cholesterol, and cLDL/cHDL ratio in the SD group, which also had higher circulating concentrations of insulin and leptin. Exercise training prevented the increase in serum concentrations of leptin, insulin, and cLDL and in cLDL/cHDL ratio. No significant differences in heart rate, systolic blood pressure, and in serum glucose, triglycerides, and free fatty acids among the groups were observed (Table 1).

**Table 1:** **Weight-related characteristics and circulating parameters**.

**Parameters**	**SS**	**SD**	**TS**	**TD**
Initial body weight (g)	25.96 ± 0.27	26.47 ± 0.30	26.4 ± 0.5	25.64 ± 0.57
End body weight (g)	30.45 ± 0.29	32.99 ± 0.45^*^	29.93 ± 0.47	29.85 ± 0.73^##^
Body weight gain (g)	4.49 ± 0.24	6.51 ± 0.36^*^	3.53 ± 0.38	4.20 ± 0.77^##^
Energy intake (kcal/day)	12.4 ± 0.67	18.59 ± 0.87^***^	12.26 ± 0.69	15.15 ± 1.25^##^^,^ ^&^
Epididymal fat (g)/body weight (g) × 1000	6.07 ± 0.46	11.01 ± 0.64^***^	5.43 ± 0.25	8.83 ± 0.74^##^^,^ ^&&^
Periaortic fat (g)/body weight (g) × 1000	0.15 ± 0.03	0.41 ± 0.06^*^	0.21 ± 0.04	0.34 ± 0.06
Systolic blood pressure (mmHg)	98.32 ± 3.19	94.45 ± 2.55	100.9 ± 4.31	96.28 ± 3.24
Heart rate (bpm)	608.4 ± 32.83	613.5 ± 19.27	585.8 ± 1.75	594.2 ± 48.87
Glucose (mg.dL^−1^)	200.3 ± 16.85	228.8 ± 24.48	181.7 ± 18.21	213.7 ± 21.11
Triglycerides (mg.dL^−1^)	87.03 ± 7.89	84.02 ± 9.23	92.93 ± 20.52	78.25 ± 11.71
Free fatty acids (mmol.L^−1^)	1.49 ± 0.08	1.80 ± 0.18	1.81 ± 0.21	1.78 ± 0.17
Total cholesterol (mg.dL^−1^)	77.45 ± 6.02	111.3 ± 8.74^**^	68.68 ± 6.87	97.96 ± 8.67^#^^,^ ^&^
cLDL (mg.dL^−1^)	32.62 ± 6.27	63.34 ± 7.46^**^	32.73 ± 4.51	47.94 ± 6.55^#^
cHDL (mg.dL^−1^)	27.47 ± 2.68	33.78 ± 3.71	21.85 ± 2.58	35.40 ± 4.67
cLDL/cHDL	1.13 ± 0.21	2.07 ± 0.32^*^	1.79 ± 0.23	1.52 ± 0.23
Leptin (ng.mL^−1^)	1.70 ± 0.21	2.96 ± 0.29^***^	1.39 ± 0.08	1.34 ± 0.24^###^
Insulin (ng.mL^−1^)	0.50 ± 0.05	0.82 ± 0.10^*^	0.54 ± 0.1	0.49 ± 0.08^#^

SS, sedentary + standard chow group; SD, sedentary + high-sugar diet group; TS, trained + standard chow group; TD, trained + high-sugar diet group. ^*^SS × SD; ^#^SD × TD; ^&^TS × TD.

* *p < 0.05*,

** *p < 0.01*,

*** p < 0.001;

# *p < 0.05*,

## *p < 0.01*,

### p < 0.001;

& p < 0.05, and

&& *p < 0.01; one-way ANOVA with Tukey post-hoc test, n = 10–18 animals per group*.

As expected, the trained groups had the highest physical performance, as evidenced by the running workload in the fatiguing exercise protocol (SS W = 0.64 ± 0.06 vs. TS W = 1.18 ± 0.09 kgm, *p* < 0.001; SD W = 0.62 ± 0.05 vs. TD W = 1.08 ± 0.06 kgm *p* < 0.001), their performance during the test was not significantly affected by the high-sugar diet.

**Figure 1:**
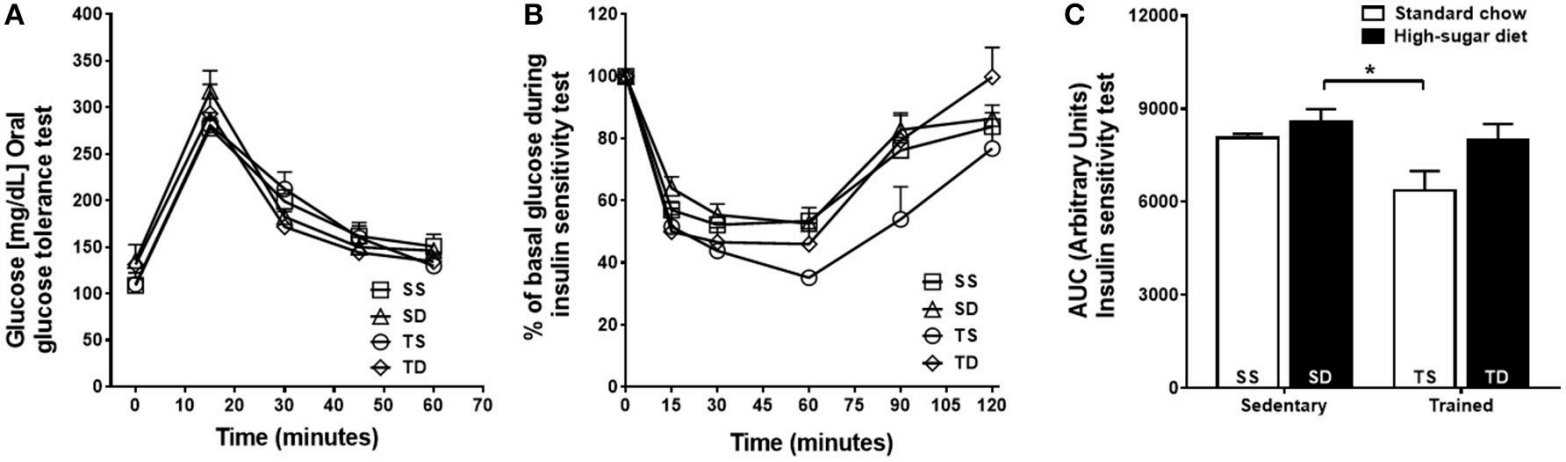
**Effects of high-sugar diet and exercise training on the glycemic profile. (A)** Blood glucose concentrations during glucose tolerance test. **(B)** Blood glucose levels during the insulin sensitivity test shown as a percentage of the initial blood glucose levels. **(C)** The area under the curve (AUC) of insulin sensitivity test. Data represent mean ± SEM. ^*^p < 0.05 SD vs. TS; two-way ANOVA with Tukey *post-hoc* test, *n* = 5 animals per group. SS, sedentary + standard chow group; SD, sedentary + high-sugar diet group; TS, trained + standard chow group; TD, trained + high-sugar diet group.

The SD group showed no significant changes in the glucose tolerance (Figure 1A) and insulin sensitivity (Figures 1B,C) tests compared with the SS group. Exercise training improved insulin sensitivity only in the TS group (Figures 1B,C).

### Vascular reactivity

**Figure 2:**
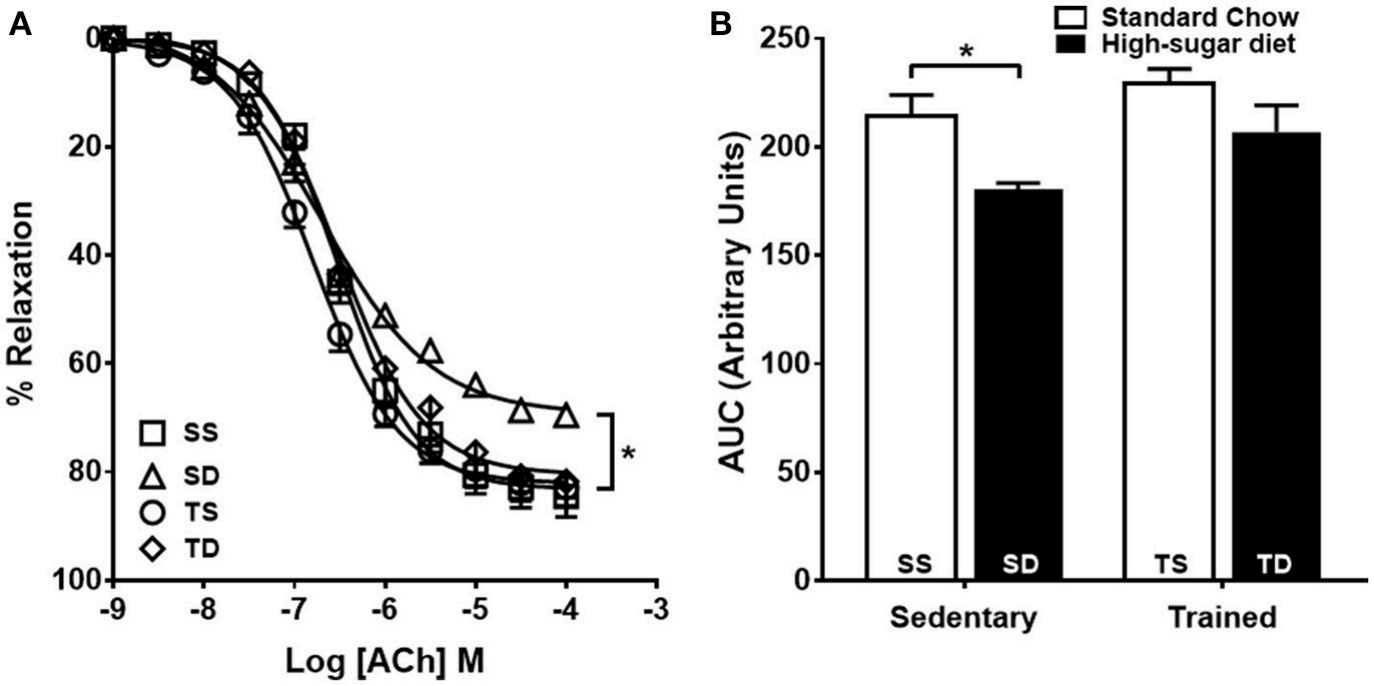
**Effects of high-sugar diet and exercise training on endothelium-dependent vasodilation. (A)** Endothelium-dependent vasodilation in response to acetylcholine (ACh), *n* = 5. **(B)** Area under the curve (AUC) calculated from **(A)**. Data represent mean ± SEM. Differences in maximal relaxation are shown in concentration-response curves. ^*^p < 0.05; two-way ANOVA with Tukey *post-hoc* test. SS, sedentary + standard chow group; SD, sedentary + high-sugar diet group; TS, trained + standard chow group; TD, trained + high-sugar diet group.

**Figure 3:**
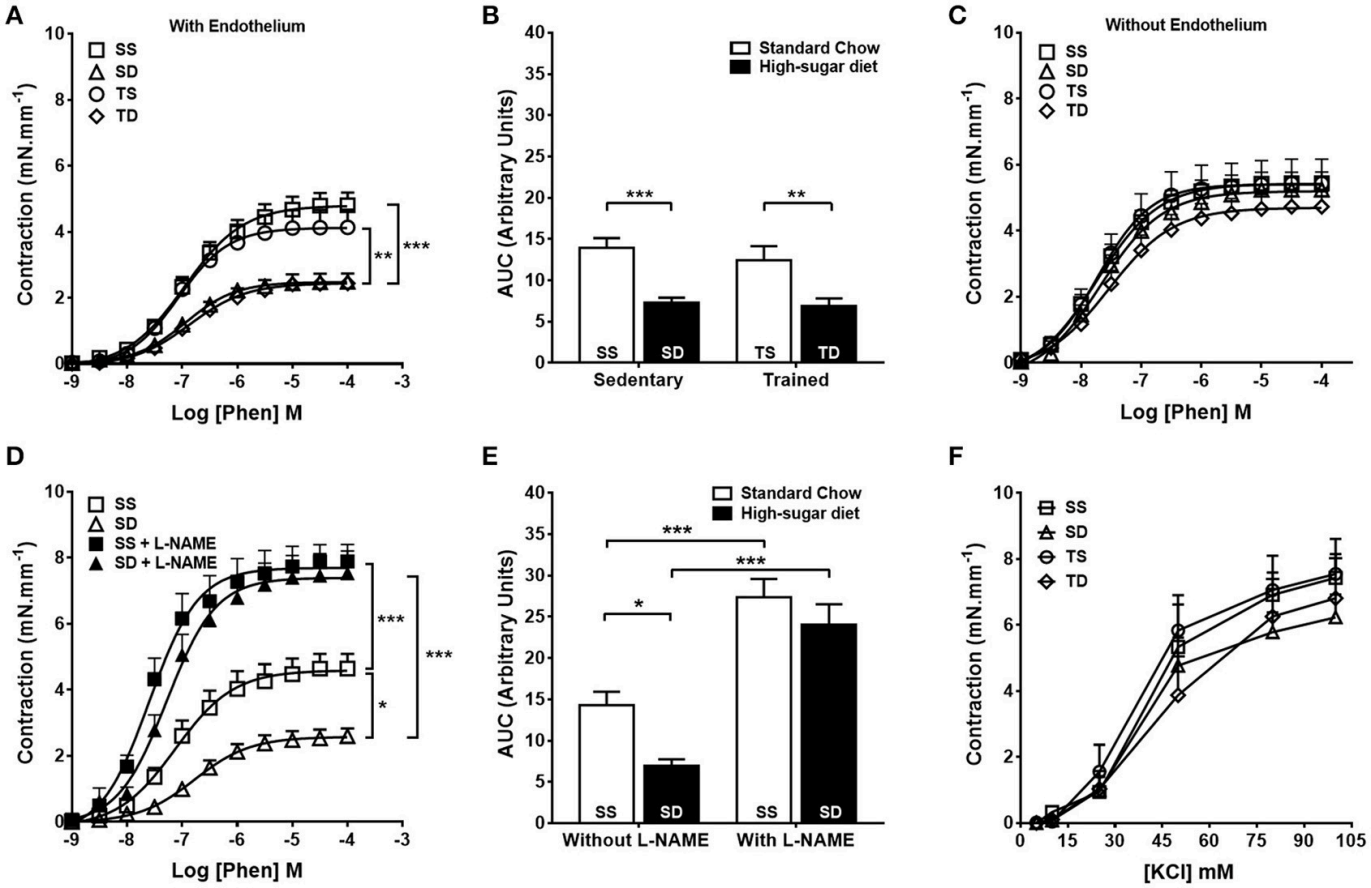
**Effects of high-sugar diet and exercise training on aorta contractility**. Concentration-dependent contraction response to phenylephrine (Phen) in the presence **(A)** or absence **(C)** of a functional endothelium, *n* = 10. **(B)** Area under the curve (AUC) calculated from **(A)**. **(D)** Contractile response to Phen in the aortas from sedentary animals in the presence or absence of L-NAME (300 μM), *n* = 8. **(E)** The area under the curve (AUC) calculated from **(D)**. **(F)** Contractile response to KCl, *n* = 5. Data represent mean ± SEM. Differences in maximal contractions are shown in concentration-response curves. ^*^p < 0.05, ^**^p < 0.01, and ^***^p < 0.001; two-way ANOVA with Tukey *post-hoc* test. SS, sedentary + standard chow group; SD, sedentary + high-sugar diet group; TS, trained + standard chow group; TD, trained + high-sugar diet group.

Endothelium-dependent vascular relaxation was impaired in the SD group when compared to the SS group, which was prevented by exercise training (Figures 2A,B). The aorta of the SD group also showed a severe reduction in the contractile response to phenylephrine (Figures 3A,B), which was normalized by endothelium removal (Figure 3C) and by L-NAME (Figures 3D,E), suggesting an endothelial NOS participation. No significant difference in the concentration-dependent contraction curve to KCl among the groups (Figure 3F) was noted, indicating that vascular smooth muscle contractility was not altered.

### Relative contribution of iNOS and eNOS to the reduction in vascular contractility in sedentary animals fed with high-sugar diet

**Figure 4:**
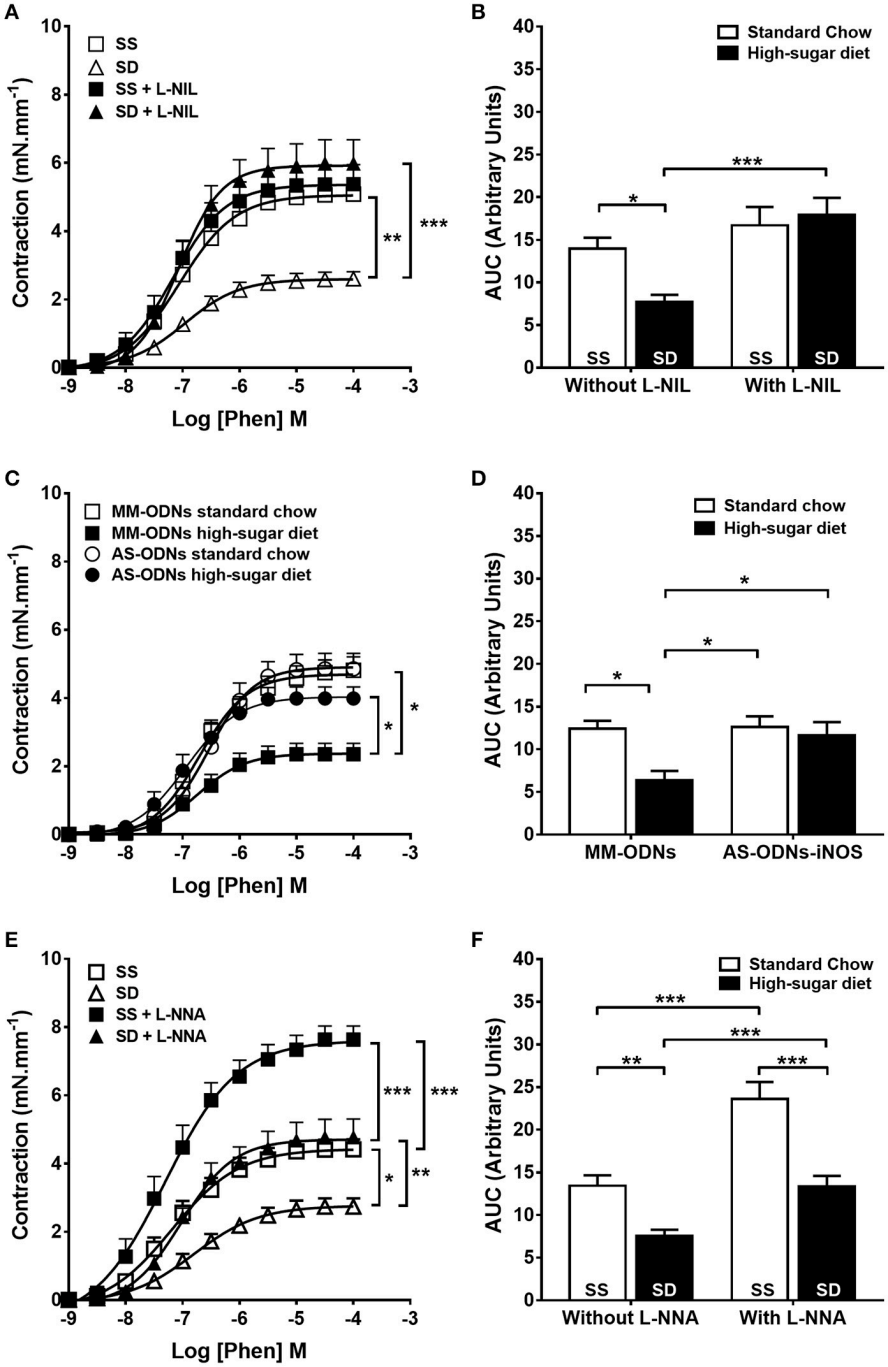
**Contractile response to phenylephrine (Phen) in sedentary animals**. The response in the absence or presence of **(A)** L-NIL (10 μM) or **(E)** L-NNA (1 μM). **(C)** Effect of antisense iNOS knockdown (AS-ODN). MM-ODNs, mismatch sequence. **(B,D,F)** The area under the curve (AUC) calculated from **(A,C,E)**, respectively, *n* = 6. Data represent mean ± SEM. Differences in maximal contractions are shown in concentration-response curves. ^*^p < 0.05, ^**^p < 0.01, and ^***^p < 0.001; two-way ANOVA with Tukey *post-hoc* test. SS, sedentary + standard chow group; SD, sedentary + high-sugar diet group; TS, trained + standard chow group; TD, trained + high-sugar diet group.

Selective pharmacological inhibition of iNOS with L-NIL (Figures 4A,B) and knock down of iNOS expression (Figures 4C,D) normalized the contractile response of the SD group to the level of controls. SD iNOS^−/−^ animals showed no alteration of aortic contractile response to phenylephrine compared with SS iNOS^−/−^ (Supplementary Figure 2). Selective eNOS inhibition with 1 μM L-NNA failed to normalize the contractile response in the SD animals (Figures 4E,F). COX inhibition did not change the reduction in contractile response to phenylephrine in SD animals (Supplementary Figure 3). Overall, our results suggest that obesity induces endothelium dysfunction and iNOS-dependent NO-mediated reduction in contractile response in mouse aortas.

### The effect of exercise training on the relative contribution of iNOS and eNOS to the reduction in vascular contraction

**Figure 5:**
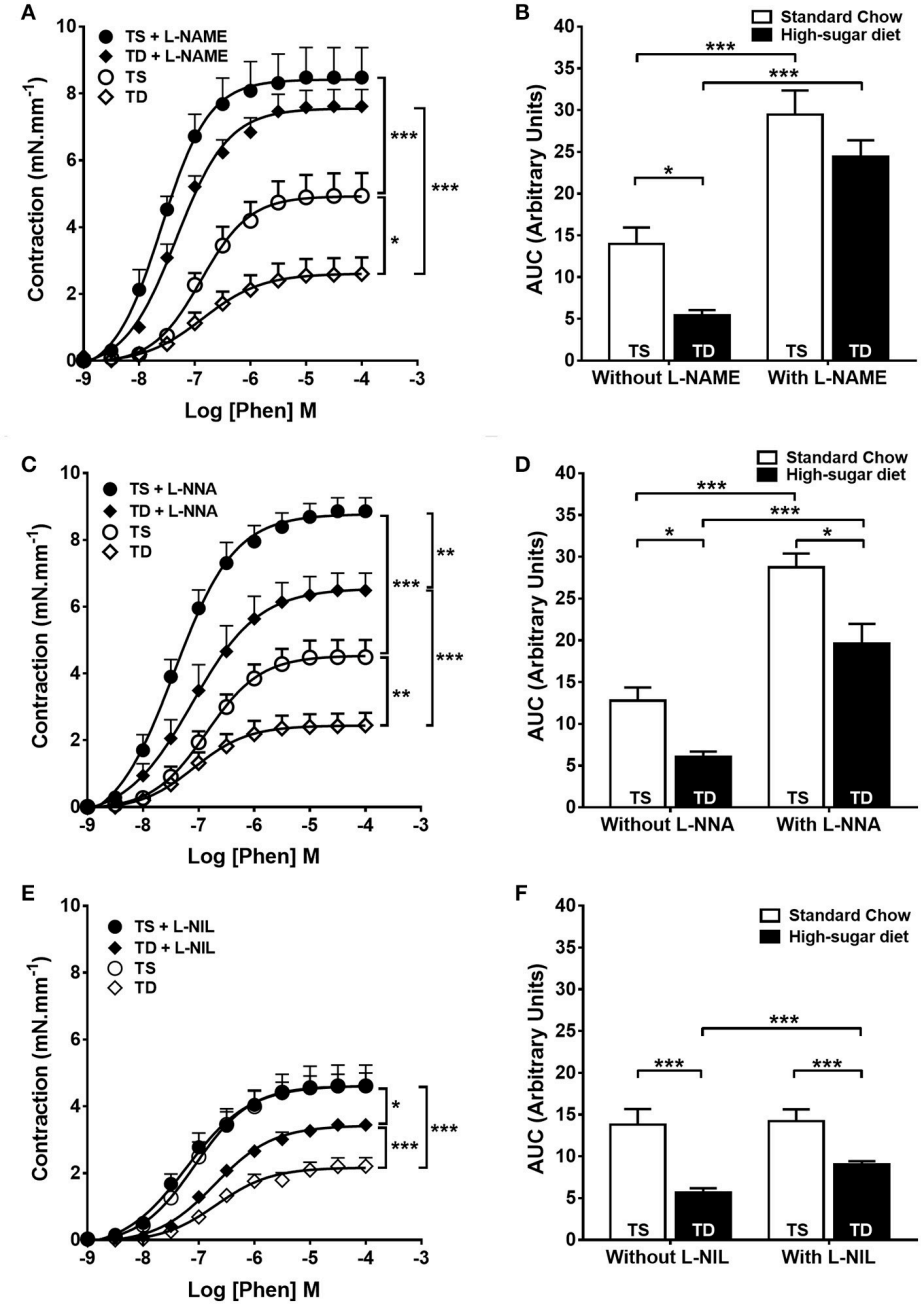
**Contractile response to phenylephrine (Phen) in trained animals**. The response in the absence or presence of **(A)** L-NAME (300 μM), *n* = 8; **(C)** L-NNA (1 μM), *n* = 8 and **(E)** L-NIL (10 μM), *n* = 6. **(B,D,F)** Area under the curve (AUC) calculated from **(A,C,E)**, respectively. Data represent mean ± SEM. Differences in maximal contractions are shown in concentration-response curves. ^*^p < 0.05, ^**^p < 0.01 and ^***^p < 0.001; two-way ANOVA with Tukey *post-hoc* test. SS, sedentary + standard chow group; SD, sedentary + high-sugar diet group; TS, trained + standard chow group; TD, trained + high-sugar diet group.

**Figure 6:**
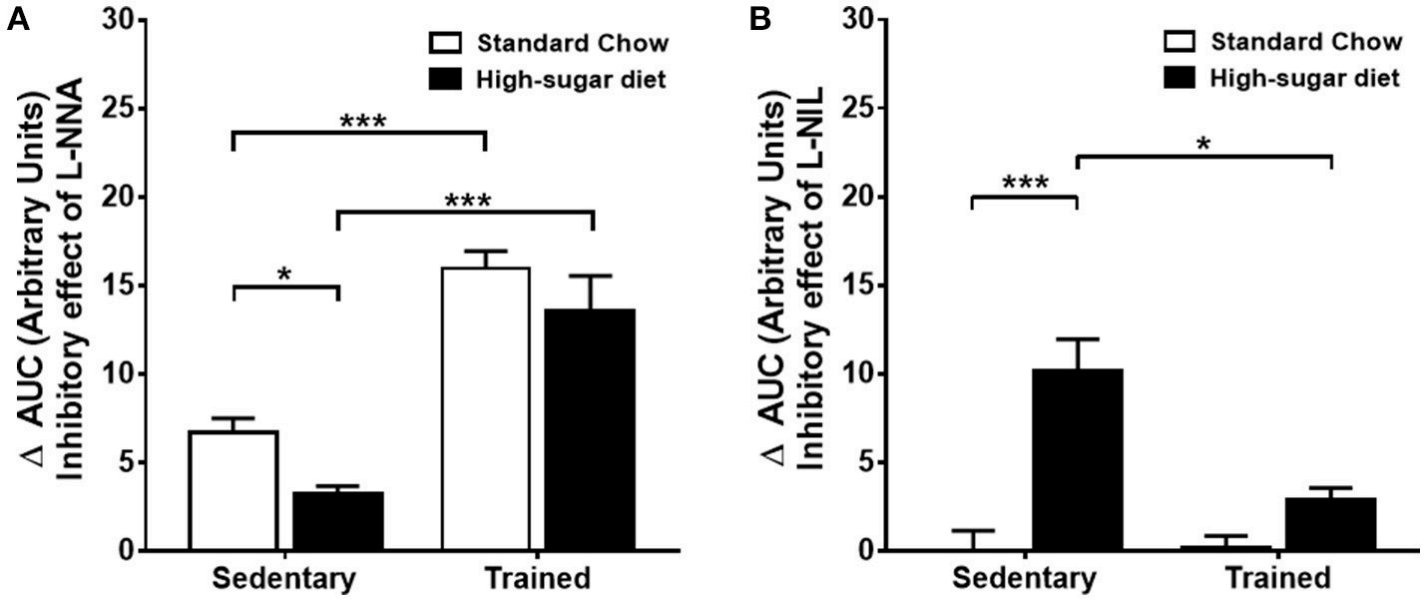
**Comparison of the inhibitory effects of L-NNA and L-NIL on the aorta of sedentary and trained mice. (A)** Delta of the area under the curve (ΔAUC) of phenylephrine (Phen)-induced contractions after and before treatment with L-NNA, *n* = 8. **(B)** ΔAUC after and before treatment with L-NIL, *n* = 6. ^*^p < 0.05 and ^***^p < 0.001; two-way ANOVA with Tukey *post-hoc* test.

The trained animals fed with high-sugar diet also showed a decrease in contractile response to phenylephrine (Figures 3A,B), which was also restored by endothelium removal (Figure 3C) and L-NAME (Figures 5A,B). Differently from the SD group, in the TD group, L-NNA partially restored the reduction in contractile response (Figures 5C,D). Additionally, L-NNA produced a greater increase in contraction induced by phenylephrine in the trained groups, compared with SD groups, as seen in the ΔAUC (Figure 6A). These results indicate that exercise increases eNOS participation in the modulation of contractile response to phenylephrine. In constrast to that found in SD group, the contractile response in the TD group was not restored by L-NIL (Figures 5E,F). The ΔAUC clearly shows that exercise attenuates iNOS participation in the reduced contractile response to phenylephrine (Figure 6B). Here, the response to phenylephrine in the trained groups was also not changed by COX inhibition (Supplementary Figure 3).

### Western blot experiments

**Figure 7:**
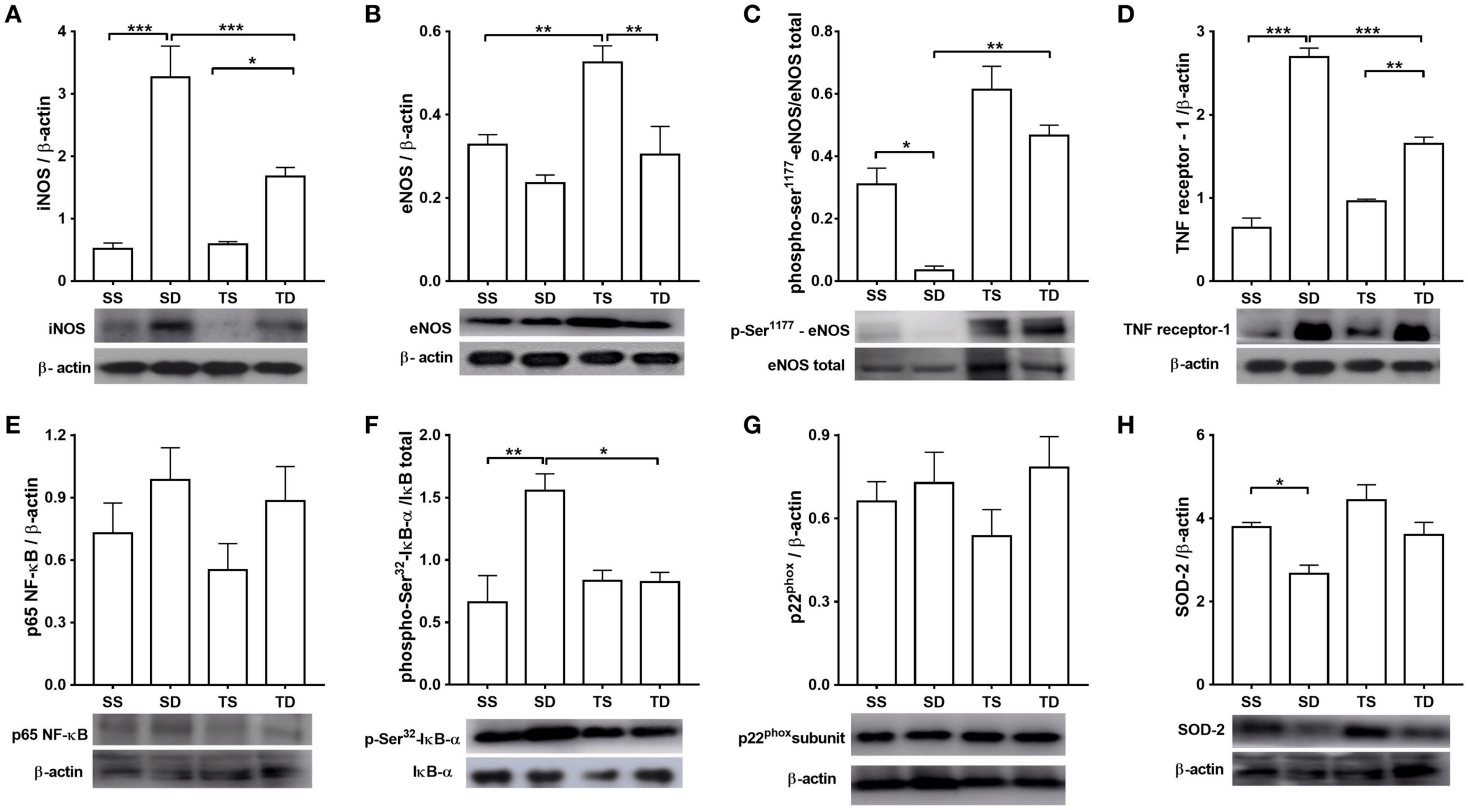
**Protein expression analyses in the mouse aorta**. **(A)** iNOS, *n* = 6; **(B)** total eNOS, *n* = 6; **(C)** phosphorylation at eNOS-Ser1177, *n* = 5; **(D)** type 1 TNF receptor, *n* = 5; **(E)** p65 NF-κB subunit, *n* = 5; **(F)** phosphorylation at Ser32 IκB-α, *n* = 5; **(G)** phosphorylation at p22^phox^, *n* = 6; and **(H)** SOD-2, *n* = 5. The histograms represent mean ± SEM. Images are representative blots from the above experiments. ^*^p < 0.05, ^**^p < 0.01, and ^***^p < 0.001, one-way ANOVA with Tukey *post-hoc* test. SS, sedentary + standard chow group; SD, sedentary + high-sugar diet group; TS, trained + standard chow group; TD, trained + high-sugar diet group.

The iNOS expression level was four times higher in the SD group than in the SS group. Exercise training attenuated the increase (Figure 7A). No difference in total eNOS expression between SD and SS group was observed; however, TS group showed an increase in total eNOS (Figure 7B). Moreover, aortas from the SD animals had a lower phosphorylation level at eNOS-ser1177 residue than those from the SS group (Figure 7C). The increase in phospho-ser1177-eNOS level shown by the TD group, compared with SD group, is noteworthy (Figure 7C). These results are in line with our functional data.

Western blot analysis also revealed that TNF receptor-1 expression was increased in the SD and TD groups compared with, respectively, the SS and TS groups (Figure 7D). However, TNF receptor-1 expression was almost 40% lower in the TD group than in the SD group. The NF-κB p65 expression was similar among the groups (Figure 7E); however, phospho-serine 32-IκB was higher in the SD group than in the SS group. Exercise training prevented the rise of IκB phosphorylation in the TD group (Figure 7F).

No difference in p22^phox^ expression among the experimental groups (Figure 7G) was noted. High-sugar diet reduced SOD-2 aortic expression in the SD group, which was not prevented by exercise (Figure 7H).

### Basal NO and superoxide production

**Figure 8:**
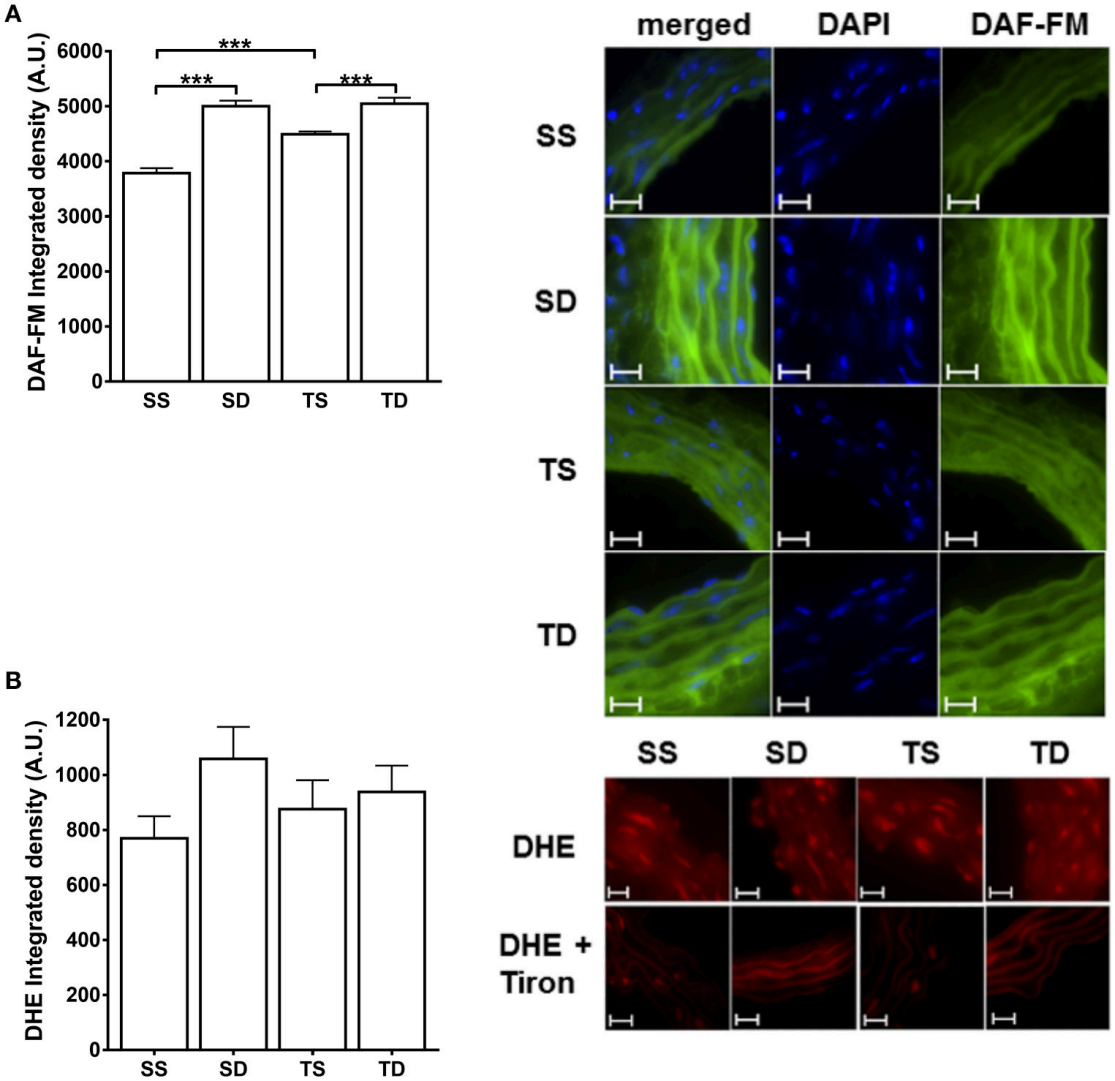
**Fluorescence detection of NO and superoxide basal production in mouse aorta**. Quantitative analysis and representative images of **(A)** NO and **(B)** superoxide anion. The nucleus stain in **(A)** was obtained with DAPI dye. The experiments in **(B)** were performed in the presence or absence of Tiron (100 μM). DAF-FM and DHE fluorescence are expressed as the intensity per vessel area. Scale bar = 20 μm. Objective 60×. The values represent mean ± SEM. ^***^p < 0.001; one-way ANOVA with Tukey *post-hoc* test, *n* = 4 animals per group. A.U., arbitrary units of fluorescence; SS, sedentary + standard chow group; SD, sedentary + high-sugar diet group; TS, trained + standard chow group; TD, trained + high-sugar diet group.

The basal NO production was increased by both the diet and exercise; DAF-emitted fluorescence was higher in the SD and TS groups (Figure 8A). No difference in superoxide aortic generation among the groups was observed (Figure 8B).

### TNFα and MCP-1 levels

The SD group showed an increase in TNFα circulating levels and fat epididymal production of MCP-1 and TNFα. The exercise training prevented these changes. No difference in the periaortic production of TNFα and MCP-1 among the four groups was noted (Table 2).

**Table 2:** **Pro- and anti-inflammatory cytokines in the serum and adipose tissues**.

**Parameters**	**SS**	**SD**	**TS**	**TD**
TNFα (pg.mL^−1^) serum	9.92 ± 3.25	37.81 ± 7.27^**^	11.30 ± 3.64	13.20 ± 3.24^##^
MCP-1 (pg.mL^−1^) serum	123.5 ± 25.09	113.0 ± 13.66	137.2 ± 25.66	87.04 ± 22.61
TNFα (pg.mL^−1^) epididymal fat	335.3 ± 71.18	1143.0 ± 15.3^***^	383.0 ± 18.5	351.8 ± 81.13^###^
MCP-1 (pg.mL^−1^) epididymal fat	70.79 ± 15.01	195.2 ± 14.73^***^	79.30 ± 16.77	67.89 ± 9.02^###^
TNFα (pg.mL^−1^) periaortic fat	1541.0 ± 191.6	1515.0 ± 32.4	1019.0 ± 30.2	1498.5 ± 84.5
MCP-1 (pg.mL^−1^) periaortic fat	58.30 ± 8.11	48.81 ± 7.19	43.25 ± 8.26	56.08 ± 11.39

SS, sedentary + standard chow group; SD, sedentary + high-sugar diet group; TS, trained + standard chow group; TD, trained + high-sugar diet group. ^*^SS × SD; ^#^SD × TD.

** *p < 0.01*,

*** p < 0.001;

## p < 0.01, and

### *p < 0.001; one-way ANOVA with Tukey post-hoc test, n = 8 animals per group*.

## Discussion

The main findings of this study are as follows: (1) iNOS has a noteworthy role in obesity-related vascular dysfunction in a model without comorbidities; (2) exercise training decreases the inflammatory state in high-sugar-fed animals, indicating a change in the relative contribution of iNOS and eNOS in controlling mouse aorta response.

Obesity is characterized by an expanded and dysfunctional adipose tissue besides metabolic changes that predispose the individuals to develop other disorders, such as hypertension, insulin resistance, and type 2 diabetes (Hajer et al., 2008). In this study, the SD group showed features consistent with obesity development, i.e., significant increase in body weight, fat mass, adipose tissue inflammation, and serum total cholesterol, cLDL, leptin and insulin concentrations, as described previously (Hartvigsen et al., 2007; de Lima et al., 2008; Hafstad et al., 2013). Although a strict relationship between obesity and cardiovascular and/or metabolic diseases exists (Jonk et al., 2007; Kobayasi et al., 2010; Campia et al., 2012), our obese mice showed no significant differences in systolic blood pressure, heart rate, glucose tolerance, and insulin sensitivity, albeit the insulin level was higher in the SD group. This potentially due to greater energy carbohydrates intake. Thus, the high-sugar diet used in this study induced obesity without other comorbidities, such as diabetes or hypertension.

A consensus in the literature that exercise training induces weight loss and improves physical performance and glucose and fat metabolism (Donley et al., 2014; Liu et al., 2015; Shen et al., 2015) exists. Indeed, we observed body weight normalization and a significant adipose tissue pad mass reduction in the TD group, showing that exercise training prevents obesity development. Furthermore, both trained groups showed increased physical performance. Of interest, the training capacity in TD group, compared with the TS group, was not affected by the diet. This result can be attributed to the training protocol performed simultaneously with 8 weeks of high-sugar overfeeding.

Similar to other reports (Yang and Chen, 2003; Marchesi et al., 2009; Deng et al., 2010; Kobayasi et al., 2010), we found a reduced acetylcholine-induced vasodilatation in the aorta of the SD group. This endothelium dysfunction is probably associated with impairment in eNOS-derived NO production, since the phosphorylation level at the eNOS serine1177 activating site was decreased, without modification in total eNOS expression. On the other hand, exercise training induced a significant rise in total eNOS expression in the aorta of the TS group and no reduction at the phospho-ser1177-eNOS level. These results corroborate our functional experiments in that the acetylcholine-induced relaxation response of the TD group was not different from that of the SS and TS groups. These findings are consistent with other studies showing that exercise training can improve the vascular function and stimulate the NO/eNOS pathway (Liu H. et al., 2015; Wang et al., 2015).

Increased endogenous free radical formation is present in obesity development and can contribute to oxidative stress and endothelium dysfunction in the aorta of obese animals (Marchesi et al., 2009; Kobayasi et al., 2010). Oxidative stress could be related to the greater activity of NADPH oxidase (NOX), a major source of ROS. However, in our study, we observed no change in p22^phox^ expression, an important regulatory NOX subunit. Furthermore, no difference in superoxide levels among the groups, despite a decrease in SOD-2 expression in the SD group, was observed. Contrary to other obesity models, the obese animals in this study showed no oxidative stress. This difference could be associated with the absence of obesity-related diseases, e.g., diabetes and hypertension, usually found in the majority of obesity models (Viswanad et al., 2006; Kobayasi et al., 2010).

Endothelial dysfunction could lead to enhanced vasoconstriction, resulting in a decrease of arterial compliance, which is observed in obesity and hypertension (Viswanad et al., 2006; Cho et al., 2011; Liu H. et al., 2015). However, in our study, the SD group showed a reduced contraction response to phenylephrine due to the increase in basal iNOS-derived NO production, since the selective pharmacological inhibition of iNOS and iNOS knock-down normalized the contraction. Moreover, in iNOS^−/−^, the reduction in contraction was not observed. The increase in iNOS expression and NO overproduction in the aortas in the SD group are in line with the above hypothesis. Together, these findings indicate an important role of iNOS in the modulation of contractile response in the aorta of obese animals. Moreover, this may represent a compensatory mechanism related to obesity in the absence of other comorbidities, which allows aortic compliance improvement in response to a possibly increased blood flow to nourish growing tissues.

The phenylephrine-induced contractile response was also reduced in the TD group; however, iNOS-derived NO only had a minor participation. This hypothesis is supported by the following results: (1) selective iNOS inhibition partially normalized contractile response and (2) exercise attenuated iNOS expression induced by the diet, which is similar to the finding by Yang and Chen (2003), that exercise training reduced iNOS expression in the hypercholesterolemic rabbit aorta (Yang and Chen, 2003).

Exercise training also raised eNOS participation in the modulation of contractile response in the trained groups, which is related to the increase in the phosphorylation level at ser1177-eNOS in the TD group aorta and vasodilator response improvement. Moreover, selective pharmacological inhibition of eNOS tended to normalize contractile response in the TD group. Finally, an increase in total eNOS expression in the TS group was observed.

COX-derivate, prostaglandins, and thromboxane are potent vasoconstrictors that have a significant role in regulating vascular function (Félétou et al., 2011). Diabetes and other obesity-related diseases are frequently associated with abnormalities in vascular reactivity related to alterations in COX-derivative production (Guo et al., 2005). Here, the reduction in contractile response in the SD animals was not dependent on COX pathway, as observed in the functional experiments in the presence of ibuprofen, which is in contrast to that observed in other studies (Meyer et al., 2013). This difference could be related to the absence of obesity-associated comorbidities in the model used in this study.

Obesity is considered a chronic low-grade inflammation state with increased pro-inflammatory cytokine, e.g., TNFα (Galic et al., 2010). Previous studies reported that TNFα pathway activation in inflammatory disorders can induce iNOS and other pro-inflammatory protein expressions (Sack, 2002; Gómez-Hernández et al., 2014). In our study, we observed that obese animals had a greater TNFα circulating level and an increase of TNF receptor-1 in the aorta. Furthermore, our results suggest that the nutritional excess induced systemic inflammation in the SD group, which led to a vascular inflammatory response, consequently inducing iNOS and TNF receptor-1 expressions. TNF receptor activation may stimulate NF-κB signaling, resulting in transcriptional events (Sack, 2002). Some studies have demonstrated that NF-κB is regulated mainly by phosphorylation of inhibitory proteins, the IκBs, which retain NF-κB in the cytoplasm (Baud and Karin, 2001; Arkan et al., 2005). TNFα/TNF receptor signaling can activate kinases, e.g., IKβ kinase, which leads to IκB degradation, resulting in the induction of NF-κB DNA binding activity (Baud and Karin, 2001). In this study, although, a difference in the p65 subunit of NF-κB expression was not found, the inhibition of this nuclear transcription factor was lower in the SD group than in the SS group; an increase in IκB phosphorylation in the obese animals was observed. In the TD group, no increase in IκB phosphorylation was noted, and type 1 TNF receptor expression was attenuated. These results may explain the decrease in iNOS expression resulting from the exercise.

In conclusion, our results show that up-regulation of iNOS induced by obesity-related pro-inflammatory factors modulates aorta reactivity, which in turn leads to a reduction in the contractile response, in an obesity model without comorbidities. Furthermore, obesity induces a decrease in eNOS functioning and in endothelium-dependent vascular relaxation. Exercise training proved to be an effective non-pharmacological therapy to control iNOS-dependent NO production and to preserve endothelial function in obesity.

## Author contributions

JS performed and analyzed the overall data and participated in the writing of the manuscript. IC and PL performed the exercise training and metabolic parameters experiments. TD conducted the measurements of nitric oxide. RS, SC, and CC contributed to the conception and interpretation of data. VL contributed to the conception, design, interpretation of data and wrote the manuscript.

### Conflict of interest statement

The authors declare that the research was conducted in the absence of any commercial or financial relationships that could be construed as a potential conflict of interest.
