# Supplementary material for: Obesity, Inflammation, and Exercise Training: Relative Contribution of iNOS and eNOS in the Modulation of Vascular Function in the Mouse Aorta
Source: Front Physiol. 2016 Sep 7;7:386. doi: 10.3389/fphys.2016.00386 (PMC5013134; doi:10.3389/fphys.2016.00386)
Supplement: Supplementary file 1 [file Image1.PDF]

Supplementary Figure 1

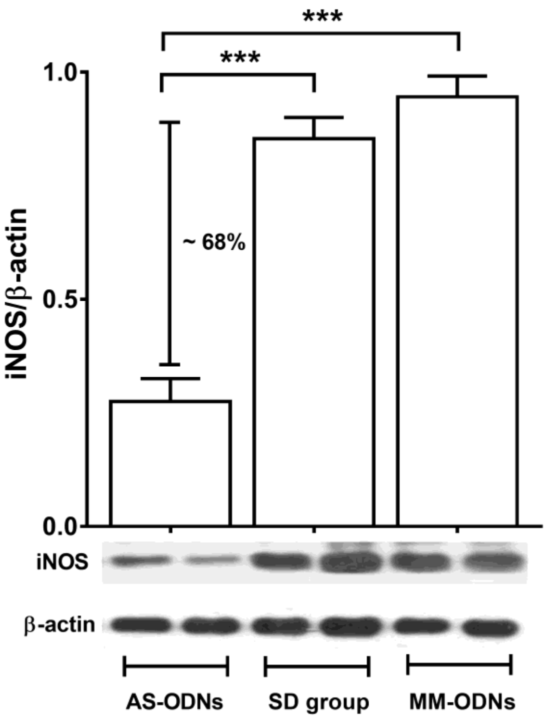

Supplementary figure 1 - Reduction of iNOS expression with antisense oligodeoxynucleotides (AS-ODNs). MM-ODNs: mismatch sequence. Data represent mean ± SEM, *** p<0.001, one-way ANOVA with Tukey *post hoc* test, *n* = four animals per group. SD: sedentary + high-sugar diet group; AS-ODNS: SD group treats with antisense oligodeoxynucleotides to iNOS mRNA; MM-ODNs: SD group treats with mismatch oligodeoxynucleotides sequence to iNOS mRNA.
