# Supplementary material for: Obesity, Inflammation, and Exercise Training: Relative Contribution of iNOS and eNOS in the Modulation of Vascular Function in the Mouse Aorta
Source: Front Physiol. 2016 Sep 7;7:386. doi: 10.3389/fphys.2016.00386 (PMC5013134; doi:10.3389/fphys.2016.00386)
Supplement: Supplementary file 2 [file Image2.PDF]

Supplementary Figure 2

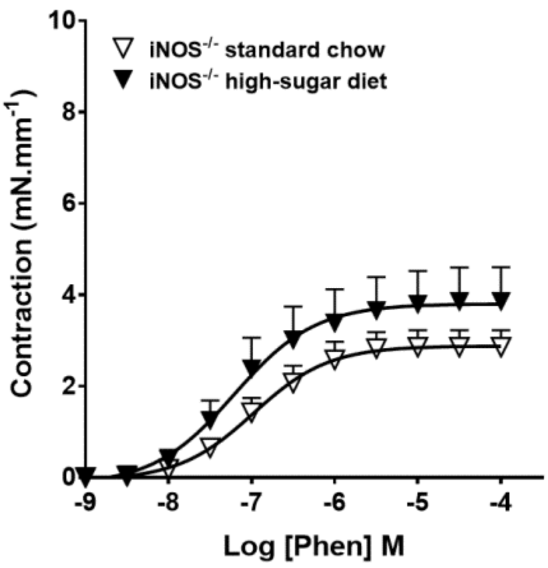

Supplementary figure 2 – Contractile response to phenylephrine (Phen) in iNOS knockout animals ($iNOS^{-/-}$) fed with standard chow or high-sugar diet. Data represent mean ± SEM, *n* = 7.
