# Supplementary material for: Obesity, Inflammation, and Exercise Training: Relative Contribution of iNOS and eNOS in the Modulation of Vascular Function in the Mouse Aorta
Source: Front Physiol. 2016 Sep 7;7:386. doi: 10.3389/fphys.2016.00386 (PMC5013134; doi:10.3389/fphys.2016.00386)
Supplement: Supplementary file 3 [file Image3.PDF]

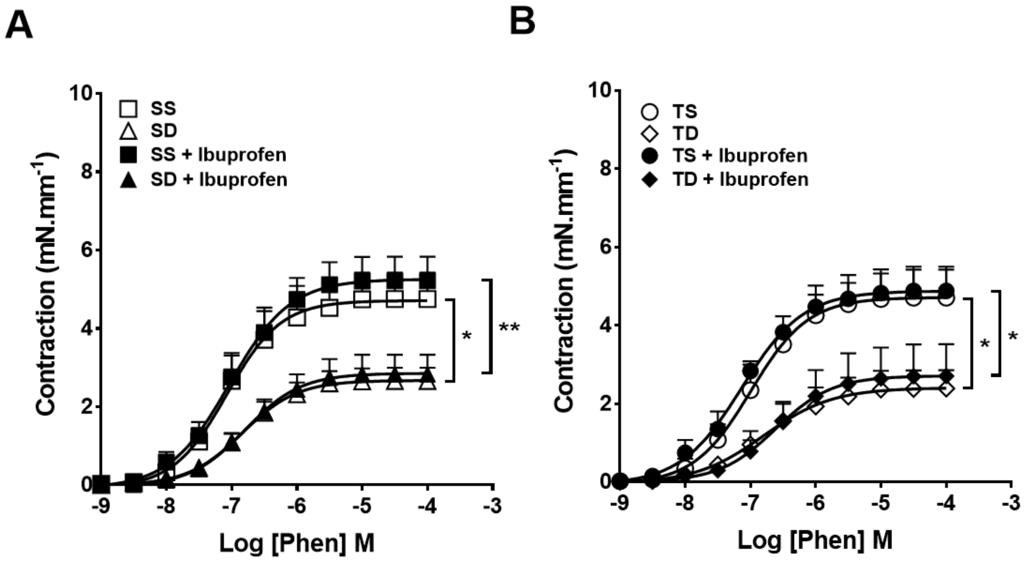

Supplementary figure 3 - Contractile response to phenylephrine (Phen) in the absence or presence of ibuprofen (10 µM) in aortic rings of sedentary (A) and trained groups (B). Data represent mean ± SEM, $n = 6$. Differences in maximal contractions are shown in concentration-response curves. *$p<0.05$ and **$p<0.01$, two-way ANOVA with Tukey *post hoc* test. SS: sedentary + standard chow group; SD: sedentary + high-sugar diet group; TS: trained + standard chow group; TD: trained + high-sugar diet group.
